# Perceived Public Participation and Health Delivery in Local Government Districts in Uganda

**DOI:** 10.3390/ijerph21070820

**Published:** 2024-06-23

**Authors:** Alex Kihehere Mukiga, Evans Sakyi Boadu, Tayebwa Edson

**Affiliations:** 1 Centre for Development Support, University of Free State, Bloemfontein 9300, South Africa; 2 School of Governance, University of the Witwatersrand, Johannesburg 2050, South Africa; evans.boadu1@wits.ac.za; 3 School of Sustainable Development, University of Environment and Sustainable Development (UESD), Somanya 00233, Ghana; 4 Department of Surgery, Mbarara University of Science and Technology, Mbarara 1410, Uganda

**Keywords:** perceptions, citizen participation, health delivery, local government, Uganda

## Abstract

Citizen participation is a crucial aspect of the national health system, empowering individuals to contribute to improving local health services through Health Committees (HCs). HCs promote the participation of citizens in the delivery of primary healthcare services. The study explores the perceptions of citizen participation in the context of the Ruhama County Ntungamo local government area, Uganda. This study aims to understand the impact of HCs on healthcare service delivery. Using a qualitative approach of inquiry grounded in thematic analysis and rooted in principal–agent theory in a single case study, this study examined citizens’ participation in the delivery of a local healthcare service. The study is based on interviews with 66 participants comprising health workers, patients, residents, health administrators, local councillors, and HC members. The findings reveal a notable absence of a health committee in healthcare delivery in Ruhama County. The absence is attributed to a need for a formalised citizen participation structure in managing health facilities and service delivery. It raises concerns about the limited influence of citizens in shaping healthcare policies and decision-making processes. The study recommends the incorporation of health committees into the local health systems to enhance participation and grant communities greater influence over the management of health facilities and service delivery. Incorporating health committees into local health systems strengthens citizen participation and leads to more effective and sustainable healthcare services aligned with people’s needs and preferences. Integrating health committees within Itojo Hospital and similar facilities can grant citizens a meaningful role in shaping the future of their healthcare.

## 1. Introduction

Citizen participation is a crucial aspect of the national health system, empowering individuals to contribute to improving local health services through Health Committees (HCs). The discourse surrounding health system governance has sparked a surge in international reform initiatives in recent decades. This process of change has involved the delegation of powers to lower levels of governance, empowering citizen participation. This initiative has led to the adoption of decentralisation policies aimed at improving service delivery [1]. The idea behind the devolution of power was to transfer service delivery responsibility from a distant central authority to local governments [2]. This would encourage more involvement from the local populace, who are better positioned to monitor service provision. Particularly, debates on progress in health have emphasised the crucial role of citizens’ involvement in healthcare delivery [3]. Healthcare delivery in Uganda and other parts of Africa has faced ongoing challenges, primarily due to insufficient allocation of resources, a fragmented and weak healthcare delivery system, and ineffective managerial decision-making [3,4,5,6]. While health committees cannot directly tackle these issues, they can serve as a vital interface for advocating necessary changes.

The Ugandan Government initiated participation frameworks in what is viewed as one of the most ambitious decentralisation programmes that encourage the participation of citizens. These changes were also part of the new Structural Adjustment Programmes (SAPs) of the 1980s [7]. Some reforms were intended to “improve the delivery of primary health care services” in the districts [8], p. 97.

Through decentralisation, the government aimed to ensure equity and promote community participation in the health sector. This move led to the creation of many districts to increase efficiency and better service delivery. However, studies reveal that service delivery in decentralised units remains a major challenge [4,9]. Healthcare, a central focus of this paper, is regarded as a basic human right. In this regard, achieving this fundamental human right requires the inclusion of beneficiaries in the development and administration of health facilities. Thus, citizen participation is an important part of achieving universal health coverage [10]. Within the framework of Uganda and this paper, citizen participation is understood to be the active engagement of citizens in the development and management of public health facilities through the community participation principle of Health For All (HFA) and Health Committees (HCs) [11,12,13]. HCs facilitate social accountability through engagements with health providers on the quality of health, challenges, and failures [9,11]. 

In this paper, we analyse the perceived participation in local health units and the outcomes in health delivery. We answer the following questions: How is participation promoted in health delivery? How do citizens perceive their participation? What are the challenges? And how does participation contribute to health delivery? Historically, the system of colonisation ripped off the citizens’ right to plan and make decisions on matters that affect their everyday lives [4]. The 1997 Local Government Act decentralised the decision-making power closer to the people in a move to improve healthcare services [14]. Decentralised healthcare facilities are funded by the Ministry of Health Government of Uganda [15,16]. There was part of the shift towards decentralisation of power and administration in developing economies. This involved procurement, identifying beneficiaries, and selecting local units or community representatives instead of central ministries [15], p. 46. Nevertheless, Onzima contends that insufficient evidence exists to support the notion of accountability and underscores the limited authority bestowed upon local governments [16,17]. In this paper, we present decentralisation as the precondition that aids inclusive participation by delegating power to the lower governance units in the health sector [18,19].

The mandate to deliver healthcare services is given to the health workers, who must execute this objective involving the beneficiaries. Using the principal–agent relationship to understand how the participation of citizens in health delivery is promoted, this article analyses the perceptions of citizens’ involvement in the health committee board of Itojo Hospital Ntungamo district and service delivery outcomes. The article has five sections. Firstly, we present the concepts of participatory planning and the health system. The second section discusses the challenges in health service delivery. In the third section, the paper presents the theoretical framework for analysing HCs and health providers. The fourth section presents the methods used in data collection. The final section presents the results and discusses the study findings. 

## 2. Global Health Governance 

Global health governance involves the management of health on an international scale, recognising the interconnectedness of nations and the need for cooperation to address global health issues [20]. However, the recent COVID-19 pandemic underscored health inequalities within global systems, particularly in vaccine production and delivery to developing nations, specifically African countries. This underscores the intersection of health governance with human rights, particularly the right to life, making it crucial for health to remain a top priority for all nations, irrespective of their economic status. The evident discrepancies in healthcare quality and service provision between developed and developing countries further highlight the importance of addressing global health governance. Health sector governance is crucial for effective service provision. Good governance includes stakeholder input, accountability, political stability, government efficiency, regulatory quality, adherence to the rule of law, and the prevention of corruption [20]. Despite extensive global debates on the need to strengthen health systems in low-income countries, less attention has been given to healthcare governance and its essential role in resource management within resource-constrained environments.

As a result, the delivery of quality health services in Sub-Saharan African nations continues to face significant challenges despite financial commitments from both local and foreign sources [21]. Issues such as inadequate service delivery, mismanagement, corruption, and substandard health infrastructure persist. Progress in health indicators has been slow, with only a slight drop in infant mortality rates from 107 to 66 per 1000 live births between 1990 and 2013 [22]. Limited access to quality healthcare, weak human and institutional capacities, and insufficient government funding further exacerbate the situation. Institutional capacities must be reinforced with stakeholder perspectives to better understand specific issues and health needs and to promote inclusive decision-making. In this regard, HCs play a key role in enabling the integration of citizens in the management and operation of health systems. Citizens are actively encouraged to participate in decentralised health services, promoting greater community involvement in healthcare decisions. 

## 3. Decentralised Health Policy

Decentralised health policy has gained significant attention in recent years. The distribution of power is seen as a crucial element of inclusive democracy [23]. It involves transferring power and creating accountability, under the idea that smaller organisations are more responsive and accountable. Decentralisation is seen as a mechanism to address issues such as inefficiency, slow innovation, and unresponsiveness to patient needs in large, centralised public institutions. When implemented effectively, decentralisation is expected to improve fairness, effectiveness, quality, and access to healthcare services, leading to better health outcomes [24]. However, while decentralised financing has seen success, management still poses challenges. An inclusive approach to managing health services is crucial for gaining a deeper insight into patients’ values and preferences. This understanding is essential for delivering high-quality health services [25]. 

## 4. Participation Planning and Health System

Participation planning includes a diverse network of actors who need inclusion and guidance through public engagements by professionals [17,20,21]. In the late 1970s, international health policies emphasised the importance of community participation in improving health delivery in developing countries [12]. Engagement of communities has been advocated for many years as a strategy to improve the health system’s performance. A study on ‘universal health coverage’ recommends that the application of primary healthcare must prioritise inclusive and collaborative efforts, involving the community, promoting social determinants of health, and facilitating community development [18]. The common mechanism used to engage communities in health services is through the health facility committee [12]. However, the campaign to achieve universal health coverage has not won yet and requires social participation that considers all stakeholders [10]. A recent study suggests that the health system needs to shift the paradigm to citizen participation to improve efficiency and the quality of life for the citizens [19]. The engagement of citizens also helps in handling systems risk through acceptance and loyalty [20]. In this context, participation ensures people’s support and trust, helping manage potential system problems.

Health is defined as “a state of complete physical, mental and social well-being and not merely the absence of disease or infirmity” [21], p. 1, highlighting the importance of citizen engagement in health promotions. This importance is captured in the vision and mission of the WHO Constitution in addressing community problems. The World Development Agenda recognizes the right to health and a better environment [22]. This view is affirmed by the WHO’s call for countries to focus more on the issue of health in their national policy agendas. 

## 5. Health Service Delivery Challenges in Sub-Saharan Africa

The challenges of health service delivery are intimately linked to the institutional health management framework. A recent comparative study of HCs’ experience in Uganda and South Africa shows that most of these committees are not seen as part of management [4]. However, the study shows that HCs in South Africa understand the role of taking part in managing health facilities than their Ugandan counterparts. In an ambitious move in 1997, the Ugandan government decentralised the delivery of health services to enhance citizen participation [14]. However, health delivery remains a huge challenge across districts’ health units. Healthcare services are in poor condition, resulting in people dying of avertable diseases [14]. The recent health delivery review on the Ugandan health system suggests there are inequalities in health delivery [14]. Studies on World Development estimates suggest severe inequalities in geographical patterns of the countries in the global South. The persistence of high levels of inequality in Southern Africa is partly attributed to historical determinants, such as the geographical pattern that reveals the shadow of colonial settlers. In this regard, most of the global efforts have been focused on strengthening local governance through the involvement of people in health planning [23]. 

The progress in the sustainable development agenda shows some difficulties in achieving healthcare goals, particularly in the lower units of government [24,25,26]. Fostering ahead toward the realisation of these goals, the UNDP service delivery framework has established a hotspot approach to local development planning, prioritising service delivery. The UNDP Malawi adopted a district’s Hotspots Dashboard that ‘enables district councils to collect and evaluate data and coordinate service delivery based on real-time information’. The idea is to ensure better targets and prioritise hotspot areas where people risk being left out of service delivery. In this regard, achieving SDGs in Sub-Saharan Africa requires addressing gaps in health delivery. 

The structural adjustment programmes in the early 1980s introduced some health reforms promoting accountable health systems. HCs became one of the ways through which citizens can participate in planning and decision-making on health delivery [4]. For instance, the late 1980s decentralisation reforms in Uganda were seen as an attempt to bring the decision-making power closer to the people. The government initiated a citizen participation approach to solve healthcare delivery challenges by implementing extensive public sector decentralisation as observed by Batley and Mcloughlin [27] and implementing a new national health policy. However, it has been argued that the success of devolution and participation strategy depends on a strong state that ensures reliable directives and “a well-informed public backed up by a participatory political culture” [28], p. 165.

IRIS Centre University of Maryland study states that Uganda facilitated citizen participation at the district level in a decentralisation process grounded on the following principle: “The better the matching, the more responsive can and will the government be” [29], p. 1. The principle seeks to understand whether officials at the local level are aware of the people’s preferences compared to officials in the central government. 

## 6. Principal Agent Theory

In this article, we apply the principal–agent theory to elucidate the decision-making processes involved in health service delivery, particularly those determined by leaders [30]. Economists and political scientists have widely utilised this theory to evaluate intergovernmental allocations [29,30]. Researchers have applied the theory to examine the relationship between healthcare providers and patients. Accountants have applied the theory to explain contractual relationships of private parties like landlords and tenants or businesses and managers [31,32]. The contractual relationship in principal–agent theory offers an analysis frame to explain citizen participation in decentralised health units through HCs. 

Within a contractual relationship, the principal, an individual, organisation, or institution with specific objectives, relies on an agent to execute plans to accomplish set objectives. In this contractual relationship, agents may have their interests, even when they share common objectives with the principal, like personal gain or self-promotion. Given that agents possess specialised knowledge and expertise as street-level bureaucrats [33], they hold an advantageous position that allows them to prioritise their interests over those of the principal. Obtaining information asymmetry often incurs high costs, making it difficult for principals to access such information [31,34]. Consequently, the principal must shape incentives that match with the agents’ interest to pursue their objectives. Furthermore, the principal employs tight supervision and penalties to ensure the agent undertakes actions aligned with the set objectives. In most models, there is an assumption that the principal reaps the profits generated by the agent. Apart from information asymmetry, this theory further addresses matters related to monitoring and information control [31,35].

Applying the principal–agent framework to decentralised health systems, we can interpret the Department of Health (DH) in the decentralised unit as the principal. The DH attempts to achieve the objectives of enhanced service delivery, prioritising this over profit-oriented goals typical in economic models. The health workers serve as agents entrusted with ensuring health service delivery and fulfilling the set aims. Monitoring the performance of the agreed contract entails promoting citizen participation in Health Committees (HCs), which increases awareness of available quality services and empowers individuals to address inadequate service delivery. Elected officials, such as councillors, members of parliament, local government chairpersons, and mayors, also monitor participation in decentralised health units through the local political process, working alongside the Department of Health.

The analysis of health committees introduces multiple principles due to the involvement of citizens, politicians, and the Department of Health. Performance monitoring becomes complex when health workers align themselves with politicians with expertise in their respective domains. This creates an information symmetry challenge between local politicians and knowledgeable health workers according to Batley and Mcloughlin [27], occasionally leading to deviations from procedural rules. In response, the principal may introduce direct incentives to address this asymmetry. In cases involving multiple principals, the contractual agreement is sometimes compromised, leading to alternative dynamics such as political influence and corruption, which undermine the benefits for the third principal. 

## 7. Materials and Methods 

This study employs qualitative research methods to explore the participation of people in health delivery in the local government health facility. Qualitative methods are commonly utilised in social sciences, offering insights into illness and healthcare’s social and cultural dimensions [36]. The choice of qualitative methodology is motivated by the need for close interaction with study participants to comprehend the participatory approach fully. For instance, a previous qualitative research study examining accountability across 18 local districts in Uganda found that survey-based approaches and quantitative investigation might inadequately detail government responsiveness and relational conclusions [37], pp. 1–16.

### 7.1. Case Study Approach 

Instead of relying just on external validity (i.e., generalizability), an internal and constructive validity-based case study design is chosen to investigate the study [38,39]. The research was conducted at Itojo Hospital, a district referral hospital located in the Ruhama County Ntungamo district local government area. As the general hospital in the district and one of the 43 health units, Itojo Hospital offers a suitable research setting. The study adopted a case study approach, enabling a comprehensive examination of the investigated case. This approach allows for an in-depth analysis of a particular event or incident within a broader context over a specific period [40,41].

### 7.2. Sampling Procedures and the Participants 

The participants of the study included the beneficiaries, health workers, district officials, local council representatives, and other key informants in Ruhama County within the Ntungamo local government area. The study utilised a maximum variation sampling approach to select individuals with diverse attributes, such as district authorities, health professionals, local council members, and several other dimensions [38]. We carefully chose individuals who met all the specified criteria to participate in the research. Our selection included individuals aged 18 and above who were receiving services at Itojo Hospital. District officials were chosen based on their roles in district committees, while key informants [40,42] were individuals from political parties familiar with health programmes in the district. The health workers comprised both current staff and individuals who had previously been affiliated with the hospital. The study interviewed 66 participants through electronic, telephonic, and face-to-face interviews [40,41,43,44,45].

### 7.3. Data Collection Instruments

The required information was gathered for the study using various data-gathering methods. The following is a list of the instruments used in this paper:

Semi-structured interviews: Semi-structured interview guides were carefully crafted to facilitate in-depth face-to-face interviews, allowing for rich and detailed data collection. These interviews facilitated open and two-way communication [39]. A diverse group of (48) beneficiaries took part, including (20) individuals who had recently received medical care at the health facility, (28) residents of the Ntungamo district who were well-acquainted with the health service delivery challenges at Itojo hospital, (10) health professionals, and (3) health administrators. In addition, (2) local councillors and (3) district officials were also interviewed to provide a comprehensive perspective. Throughout the interview process, a strong rapport was established, creating a supportive environment that encouraged study participants to openly share their personal experiences. 

Key Informant Interviews (KIIs): Using Kumar [42] in-depth KIIs, health administrators, local councillors, and local council members were interviewed. The technique was adopted to solicit relevant information from individuals with in-depth knowledge and perspectives on national and local health services through HC. Eighteen (13) interviews were conducted with health administrators and health workers using KII techniques. The interview data were collected using an audio recorder, and each recording lasted between 45 and 60 min. It was later transcribed into a word document for further analysis using qualitative software Atlas.ti. Two (2) local councillors and three (3) district administrators participated in KIIs.

Document analysis: The analysis incorporated the literature relevant to citizens’ participation in national and local health services through Health Committees (HCs) in Uganda to complement the field interviews. Utilising Bowen [46] thematic analysis, documentary evidence like district scorecards, policy briefs, newspaper reports, and unpublished reports on the state of health in the Ntungamo district were used to enrich the analysis. To achieve a thorough analysis of pertinent data, we meticulously selected and scrutinised 20 documents and web pages, following the criteria outlined in Table 1. This paper utilized a documentary approach to analyse the data [40,41,46,47] This rigorous examination aimed to achieve data saturation and encompass all potential information. 

## 8. Data Analysis 

Thematic analysis, as put forward by Clarke and Braun [48], was used to categorise the codes developed from data into themes. The three-step reading of each transcript and matching each transcript with the field notes served as the starting point for the coding procedure. This provided the solid groundwork for the data’s hand coding. These codes were sorted and categorised according to topics to accomplish the study’s goal. The study evaluated the study’s robustness using Lincoln and Guba’s [49] “four-dimension criteria” in qualitative research (credibility, dependability, confirmability, and transferability). Using a triangulation method [48,49,50,51,52], the interviews with beneficiaries, health workers, district officials, local councillors [53], and key informants [42] were triangulated with the documentary literature to ensure data accuracy and robustness of the interpretation [54,55]. To ensure participants’ confidentiality, the paper used pseudo-variables to present the empirical outcome, as shown in Table 2.

Participants in this study willingly gave verbal consent and agreed to have their In-Depth Interviews (IDIs) captured for the analysis. The field data were securely stored in electronic encrypted files. Participants’ identities, personal information, and names associated with their responses were excluded from the transcription used in the analysis. Preservation of privacy, confidentiality, and anonymity was ensured. 

We employed a conventional qualitative analysis of content where we coded categories derived directly and inductively from the text [48,49]. We followed the following steps, as delineated by [55], pp. 318–330.

- Prepared the raw text retrieved from the various government institutions;
- Defined the unit of analysis;
- Developed categories and a coding scheme;
- Validated the coding scheme on sample text;
- Coded the entire text;
- Assessed consistency within the codes;
- Drew conclusions in the codes;
- Finally, we reported the findings.

The analysis of the key informant and in-depth interviews took the sequence as delineated by [56], p. 240.

- Collation of field research notes and transcription of audio interviews.
- Developing data codes (logically inductive) from the field data;
- Transformation of data codes into comprehensive labels or themes;
- Organisation of themes, labels, and categories by identifying and sorting similar phrases, patterns, relationships, and commonalties or disparities;
- The fragmented categories and labels that were sorted and scrutinised into meaningful and manageable transcript to segment patterns, thoughts, and processes; and
- The segmented patterns identified in the transcripts were carefully interpreted juxtaposing them to previous studies, theories, and frameworks to construct some level of generalisations.

To ensure data trustworthiness and validity, we adopted Lincoln and Guba’s four-dimension criteria to evaluate the interpretation and findings of the research work: credibility, transferability, dependability, and confirmability [49,55]. For credibility, we thoroughly engaged the text, triangulated it with the field data, and checked the interpretations against raw data, peer debriefing, and member-checking [49]. With transferability, although our working propositions are context-specific when applied in another context, the findings may be similar. To ensure dependability and confirmability, we checked the consistency of the field processes to ensure that the research is coherent in terms of the data, the findings, the interpretations, and recommendations [57]. 

## 9. Findings and Discussion

### 9.1. Perceived Citizen Participation in Health Delivery 

**Figure 1:**
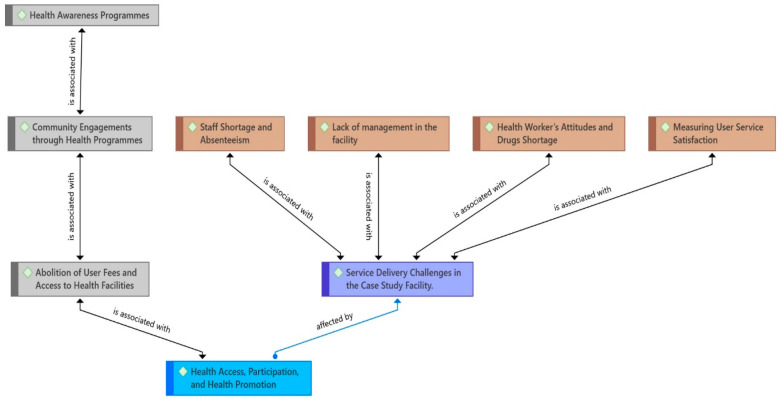
Study findings show service delivery challenges and efforts toward health promotion. Source: Created by Authors from the field data, 2024.

The perceptions of the participants on the process of citizen participation were quite telling regarding the participation of HCs. The findings suggest some discrepancies in how participants perceive citizen participatory processes. Some new themes that emerged from the data resonated with the challenges affecting healthy service delivery in district health units (Figure 1). It indicates that health delivery is affected by perceived power relations, for example, on the question of who has the decision-making power to influence the delivery of services. Most participants believed that their participation in choosing a majority leader gives them influence over those who make decisions. 

However, though participants claimed to have some influence in decision-making through choosing their leaders, the data suggest a lack of checks and balances to assess the underperformance and poor service delivery. The question about accountability through constitutional provision for the recall of leaders revealed some loopholes in the governance system that ensures consistent regulation. The constitution allows citizens to participate in health by choosing representatives who become part of the HCs. The constitution also gives citizens the power to withdraw or recall underperforming leaders from the council and parliament. However, the participants’ (beneficiaries/patients) responses suggest recalling a leader is not an easy process. One of the health beneficiaries from the district said:

*It has never happened here, and I don’t think it is possible to happen; they need a long process to do it, which is hard for common people, but even those who would wish to do it get scared because they don’t know whether people will cooperate*

(HB).

Nonetheless, it was revealed that there is an open platform, such as community gatherings. Community leaders, non-governmental organisations, councillors, and members of parliament often organise these. People can voice their grievances on these local platforms, including health service delivery and general governance systems. Leaders, including HCs, use these platforms to share their views on the district’s health and other related issues. 

### 9.2. Health Access, Participation and Health Promotion

#### 9.2.1. Abolition of User Fees and Access to Health Facilities

The decision to abolish user fees in public health facilities was a move by the government to improve access to affordable health care. To some extent, this move was influenced by increased political pressure, where opposition politicians advocated for people’s access to health facilities. The rise of Rtrd Col. Dr Kizza Besigye as President Museven’s challenger in the 2001 general elections mounted pressure that changed the health policy direction in Uganda [50]. While on a political campaign, the president announced the removal of hospital user fees, which meant that a certain wing was created for free access to the public. However, the participants stressed that free access to affordable health care is meaningless due to a lack of services at the facility. The interview with one of the general practitioners at the facility suggests that the abolition of user fees in private clinics at the hospital did not improve health delivery:

*Previously before the abolition of user fees in private clinics, the services were much better. The finances from the private clinics helped in supporting the day-to-day operation of the hospital. The allocated budget from the central government is not enough and for that reason majority of our colleagues have decided to go*

(HW).

Though the user fees were abolished as the government yielded to political pressure, service delivery is generally poor. Public hospital management relied on the user fees to run health facilities and maintain staff. The participants claim that those who receive fast services sometimes offer some money to the health workers. The claim is that patients still pay user fees indirectly to health workers. As a result of small budget allocation, the hospital often runs short of fuel for ambulances and to power the generator during load-shedding. In this instance, patients are told to buy fuel in an emergency requiring an ambulance or an operation that needs a generator. The HW further said that in most cases patients are forced to buy painkillers from the private clinics after the operation. The HW further said that after the operation, some patients wait two days to receive painkillers from the hospital nurses and one gets lucky to receive them. These issues are well managed when the people are part and partial of health management and implementation of a health access plan.

#### 9.2.2. Community Engagements through Health Programmes

The officials believe that the interactions between local leaders and health workers help reach health targets. Community awareness is achieved through the collaboration of different role players utilising various channels. Community engagements mostly involve community gatherings organised at a particular site, parish, or sub-county level and other platforms like churches, radios, TV programmes, and school visits. These spaces enable the people to express their concerns to the leadership or experts but also are used to raise awareness on certain issues. Concerning the HCs, the issues that arise during these engagements are addressed by the district Social Welfare Committees (SWCs) (The SWC is appointed by the district council during the council sitting. The committee is composed of councillors who are tasked to tackle social problems affecting communities. The committee reports back to the district council quarterly (three months).) The engagement of people in communities was adopted since the constituency assembly as the National Resistance Army (NRA) decided to return power to the people. The local officials claim that community engagements have improved health conditions in general and the spread of HIV and other communicable diseases. However, the interview with the beneficiaries reveals that most of the leaders tend to disappear after being chosen and appear soliciting support for another mandate.

Health facilities are equipped with mechanisms to ensure service standards, and some of these include feedback from communities. The local officials indicated that the hospitals offer ways through which beneficiaries can give feedback on service delivery using the suggestion box. However, one of the HWs said that most beneficiaries are illiterate and often do not think about the use of a suggestion box. Even the beneficiaries were so adamant that the use of a suggestion box was not effective. They said that even when they report, things always remain the same or get worse. There is a general belief among the people that government hospitals, especially services, will never change. For this reason, most people make use of private clinics, which offer some basic treatment. In the following interview excerpt, a health administrator observed that:

*Some people with ways to raise money prefer private clinics. They do not even mind about going to a public hospital. They go to private clinics and hospitals because they believe that public hospital services are bad*

(HA).

The local officials were adamant that public health services have generally improved as opposed to what people think. When asked why people feel that there is no inclusion in hospital management, their reaction seemed to suggest that people lack awareness of how certain things work in government. The official claimed that most people lodge complaints with their local councillors and they are worked upon. The lack of awareness to demand services was attributed to low literacy levels:

*“People who live in remote areas often feel a strong connection to their community, but they may not be aware of their rights or how to ask for better services. That’s why I believe that education is crucial for everyone. People with a good education can speak up for their rights and demand better services”*

(LC).

This seems to suggest that realising universal health coverage requires attaining some level of literacy. In this regard, the achievement of universal health coverage might require a multidimensional approach.

#### 9.2.3. Health Awareness Programmes

During the interviews, the local councillors claimed that health awareness programmes are instrumental in promoting citizen participation and increased health coverage. These health orientation programmes encourage citizens to take part in non-selective health activities. The reason for improved participation is further highlighted by the councillor:

*Participation is because we have non-selective government programmes that include all genders and different groups. Here, we do not have marginalised groups, so if someone doesn’t take part, it means that they, as individuals, are naturally not developmental*

(LC).

However, from a gender perspective, health programmes seemed to be attended by mostly women as compared to men. The challenge of men not attending health programmes has been a concern to health workers who said that most counselling sessions require both partners. This seems to suggest that the unwillingness to participate in health programmes to a certain extent weakens citizens’ involvement in the decision-making process of health services. Further, the behaviour of most men has been a challenge that the district council has been grappling with for some time. The councillor indicated that men spend time in bars instead of taking part in health awareness programmes:

*Men lie behind in participation in government programmes due to their unwillingness to participate. There has always been a debate in the council on social problems, where we have found out that men spend most of their time in bars drinking*

(LC).

This suggests that communities are not fully incorporated in the management of health services. However, Uganda’s local council structure has been effective in political control and management of communities. Strong HCs would use these spaces to influence citizens’ participation in health programmes. The advancements in health promotion can be attributed to the local government officials responsible for implementing specific programmes within communities. These programmes are often part of national health campaigns conducted across various districts. Despite the existence of a hospital management committee, the study suggests that its members are inactive in fulfilling their duties and responsibilities. This might also indicate the poor health management structure, resources, and policy priorities.

### 9.3. Service Delivery Challenges in the Case Study Facility

Medical facilities in local governments face numerous challenges in delivering medical services. These challenges exist regardless of inclusive citizen participation in health programmes. Most of these challenges alluded to HWs bending the rules and regulations governing the delivery of health services. In the principal–agent relationship, the contractual agreement is often compromised by other factors like the agent’s dishonesty, using their expertise to bend the rules, or situations where there are multiple principals. This section presents the perspective of health service delivery challenges in the Ntungamo district. 

#### 9.3.1. Staff Shortage and Absenteeism

The interview with the medical officials working at Itojo Hospital indicated that the shortage of medical personnel hampers health service delivery. The challenge of staff shortage is further exacerbated by absenteeism. The HW conferred to interviewers that:

*The expected percentage of staff in the hospital is 39%, but most of the staff normally don’t report, and even when they do, they come in late and leave early. So, you can imagine, some time back, we had only one midwife who would conduct and administer 90 patients, go to the store to fetch medication, and carry out 12 deliveries daily*

(HA).

Based on the above statement, there is a need for effective health committees to help improve community participation and monitor the roles of health workers. Active HCs demand an increase in the number of staff and availability of medication in health units.

#### 9.3.2. Lack of Management in the Facility

The lack of effective management in Itojo Hospital highlighted the challenge affecting service delivery. The medical officials mentioned the lack of seriousness of the medical superintendent who oversees the daily operation of the hospital:

*Management is still a challenge here in this hospital. Our medical superintendent is the district health officer, so he sometimes comes once a week or sometimes does not. Supervision is very poor; everyone does as he pleases*

(HW).

The HW indicated that there is late delivery of drugs from the national medical stores. This makes the work of medical personnel difficult to coordinate, and it makes it difficult to deliver efficient services to patients. Medical personnel also pointed out that patients know and understand the challenges medical officers are going through, which has become part of their day-to-day realities.

The interview with the patients also showed some level of frustration regarding the delivery of health services. One of the participants stressed that there are still a lot of unfulfilled promises. Corruption at the facility was mentioned as a big problem for service delivery. The patient indicated that even those in a critical condition need to bribe medical workers for attention. The patient’s lack of money to pay the nurses for a quick response might render one helpless:

*There are so many cases of unfulfilled delivery services; a case in point is a pregnant mother who failed in a health centre here and was referred to Itojo Hospital in critical condition. As the patient approached the medics, was asked for money, and ignored until this female patient finally produced the money*

(HB).

The practice of charging patient user fees in public hospitals is illegal following its abolition by the government. However, one of the medical personnel blamed citizens for promoting bribery:

*We cannot control someone who hides and gives the money to the nurse because there are no cameras in the hospital; how can we do that?*

(HW).

Bribery for services in public hospitals demonstrates a lack of services, and patients are extorted money out of desperation. Health committees were thought to involve the public in managing health facilities and avoid corrupt practices in health units. The absence of active health committees creates a gap for opportunism. 

Instances like insufficient resources at the facility make the service delivery complex. For example, participants indicate that patients still pay for fuel ambulances and the hospital generator. This is when the patient in a critical condition requires an operation during the load shedding of hydropower. After the operation, the patient is sometimes required to buy painkillers in private clinics, some owned by health workers in public hospitals. This might be after spending two days waiting for painkillers from hospital nurses.

#### 9.3.3. Health Worker’s Attitudes and Drugs Shortage

Health workers, as more knowledgeable agents than the principal, can often bend the rules to their advantage. The principal–agent relationship further demonstrates that where the agents have more specialities in what they are doing than the principal, the latter puts them in a more advantageous position and allows them to pursue their interests at the principal’s expense. The principal might want to overcome this information asymmetry, but obtaining information has major cost effects and may be difficult. Hence, this forces the principal to pursue his objectives by shaping incentives that align with the agent’s interests. These incentives in this context are bribing health workers to get medical attention. One of the patients narrated a situation that happened to one of the patients:

*“There are many cases where people who need medical attention cannot receive it due to problems with delivery services. For example, a pregnant woman who was having difficulty with her pregnancy went to a local health centre for help but was told to go to Itojo Hospital. On arrival, the medical staff asked her for money before they would help her, even though the patient was in very bad labour pain. The patient didn’t have the money at first, but eventually found a way to pay and got the help at lastd”*

(HB).

Patients who can raise some money use it to bribe health workers to get attention. Corruption at health facilities has become a vice in public health units. The expectation of bribes means that patients who cannot afford bribes need help to get the required attention. Limited staff and poor remuneration appear to be why some health workers develop attitudes toward patients. The few staff members are overworked, and most complained of exhaustion because of work overload. Hospitals in the decentralised units struggle with finances, which means that crucial services are lacking at these facilities. Essential services such as maternity are almost non-existent at the facility. HIV patients complained of ARVS shortage, which resulted in some patients not receiving their monthly medication. 

#### 9.3.4. Measuring User Service Satisfaction

Health committees in the public health units are expected to receive feedback on patients’ satisfaction and engage the health workers in service improvements. Various mechanisms are used to get patient feedback, such as suggestion boxes and complaints directed to the area councillor. However, the availability of these mechanisms is not reflected in the data collected from the HB. The HB refuted the claim that suggestion boxes are in place to be utilised for service improvement. Most of the participants wondered as to whether the suggestion boxes were ever opened. The HB described the hospital services as providing the worst possible experience:

*When you come to this place (hospital) you are not different from someone in a court of law waiting for the death sentence.*

While describing the state of healthcare in the facility, the HB revealed that emergency services that require the use of an ambulance are non-existent. This puts pregnant mothers in a difficult situation, especially when the delivery time starts while they are on the way to the hospital. In the event of a referral, HBs hire private cars to transport patients to the hospital. It even gets more complicated when there is load-shedding and use of the generator is required. The HBs stated that these are the daily experiences well known to the leadership and do not require the suggestion box. 

The state of service delivery was complemented by the officials’ citing improvements over the years. The improvement has been registered in the increase in staffing and community awareness programmes. The HW indicated that, years back, the hospital had one midwife who administered 90 patients:

*So, you can imagine, some time back, we had only one midwife who would conduct and administer 90 patients, go to the store to fetch medication, and carry out 12 deliveries per day*

(HW).

However, during the study, the hospital was full of long lines with other patients sleeping on the floor. There was not any indication of the improvements mentioned by the officials, but the practices mentioned by the HBs were more visible. These practices show the weakness of health committees in public health units. Health committees are not properly integrated into the health system. A recent study indicates that health committee members in Kyankwazi did not view themselves as part of the health system [45]. Executing their duties as outsiders makes the monitoring process difficult and access to information gets complicated. During the study, we observed that HBs did not know health committee members, an indication of less citizen participation in hospital management. 

## 10. Discussion

Community participation in choosing leaders such as councillors and HCs gives the expectation that these leaders will oversee and monitor health delivery. The HC is composed of councillors who are on the social welfare committee and are expected to give good oversight since they are representatives of the people. The management of health facilities requires competent committees that form part of hospital management. In this regard, HC members are expected to be more actively involved in managing and delivering healthcare in the case study district. The lack of involvement in the management is largely attributed to the need for a more formalised structure and a lack of expertise in monitoring and evaluating health performances. Scholars have argued that the health committees should not be separated from the management of the hospital [51]. 

HCs must have more expertise and an understanding of the everyday running of the health facility. The onus to serve as an HC member should be based on one’s ability to understand the daily operation of the hospital facility. This helps them to perform the most necessary duties to ensure that the health facility delivers better services. The study observed that when HCs have some speciality, they help in linking the public and health workers. More often, a shortage of resources and staff is used as a scapegoat other than scrutinising the management of the health facility.

The application of principal–agent theory in the public decentralised health units suggests that the principal may find a way of ensuring that the agent carries out the set objectives using monitoring and punishments. For local officials, this happens when seeking another mandate from the people. However, the mandate relies on an effective system that respects the will of the people, which is not always the case. In this regard, the determination of HC becomes a confusing process which makes it difficult for HB to understand the HC members.

HCs within the public health systems, including those in the case study district, are mandated to receive feedback on patient satisfaction and engage the health workers on service improvements and related issues. The HCs utilise several mechanisms to get patient feedback, like a suggestion box and complaints directed to the area councillor representatives in the district council or through the council’s social welfare committee whenever they are unsatisfied with the services. The HCs maintained that these platforms help monitor and evaluate service delivery and how they have improved or otherwise. The finding is consistent with studies in other parts of Africa where authors observed that involving HCs and patients in healthcare delivery tends to help improve the various services due to the feedback [8,9,51]. However, it has been argued that street-level bureaucrats twist rules through ‘developing techniques to salvage service and decision-making values within the limits imposed on them by the work structure’, which tends to alter the idea of service delivery [52]. It gives them a position to manipulate citizens instead of offering better services.

Furthermore, there were additional public forums, including neighbourhood get-togethers and other venues for public hearings. These are frequently arranged by local authorities, non-governmental organisations, council members, and legislature members. Community members and patients have a great opportunity to air their complaints on various topics, such as the provision of healthcare services and general governance structures, on these local forums. These forums are used by leaders, including HCs, to voice opinions on matters about the district’s health and other relevant community issues. To guarantee effective and efficient service delivery, the Ugandan health system includes Health Committees (HCs) comprising various community stakeholders. These HCs collaborate closely with the district council’s social welfare committee, as Lodenstein et al. [11] observed.

The lack of active HCs at the health facility affects efforts in monitoring and evaluating health-related service delivery. For instance, the late delivery of medicine from the national medical stores is said to be a challenge and more often the national medical store blames hospitals for not putting in order claims. In some cases, these orders are not collected from the medical store, leading to the expiration of medication. These circumstances affect the service and delivery at the health facility. HCs are responsible for monitoring the availability of resources and the function of the facility [9,51]. However, the late delivery of drugs demonstrates a lack of active participation and monitoring of the HCs at the facility. Furthermore, it also demonstrates the management vacuum, which is also the responsibility of HCs to oversee the work of hospital management. In this regard, HCs must have close oversight of the day-to-day operations at the health facility in the district.

Citizens’ participation is key to patient access to health facilities within the case study district, and the central government’s abolition of the user fee further enhanced community members’ participation and access to healthcare at the facility. The abolition of the district’s ‘cash and carry’ healthcare system was championed by political pressure and other health access findings. It emerged that having free admission to medical institutions after the abolition of user fees did not ensure the provision of treatments. However, once the services were offered, patients’ inability to pay did not prevent them from using the facility, unlike before the government removed the user charge policy, as found by Nabyonga Orem et al. [58]. The finding is consistent with a study where the authors found that HC’s crucial role is to advocate and ensure that services are available to everyone, irrespective of their social, economic, and political status [11].

Nonetheless, it was revealed that most health officials left the hospital after the abolition of private clinics at the hospital to the detriment of patients. The medical official argued that the money from the private clinics helped motivate the hospital staff and supplement the small budget allocated to the hospital by the central government. It also helped support other daily hospital activities that the current budget allocation from the central government cannot cover. This revelation contradicts the findings of Nabyonga-Orem et al. [58], who claim that patients were happy with the improved quality of service after the abolition of user fees.

Moreover, patients who can afford a small amount of money bribe medical staff to receive care. One of the vices in public health units these days is corruption in healthcare facilities. Due to corruption, patients and community members who cannot afford to pay their way through tend to find it difficult to receive the necessary healthcare. It was revealed that poor pay, abolishing the user fee, and other staff-level issues seem to be the cause of some healthcare professionals’ attitudes toward patients. Most of the staff members reported feeling exhausted due to work overload, and a handful are overworked. Additionally, decentralised health units, including the case study facility, are often plagued by financial and other human resources difficulties. Consequently, the facility tends to lack several essential services, including maternity. It came to light that HIV patients had been complaining about a lack of ARVS, which is preventing some of them from receiving the necessary monthly treatment. Despite the widespread problem of corruption and mismanagement within the healthcare system, there is a lack of significant action being taken to address these issues. As a result, the quality of healthcare services provided in public health facilities has significantly declined.

The study observed that, in health beneficiaries, it is mostly women that actively participate in health orientation programmes. The council member emphasised that men do not participate fully and highlighted that this has been a challenge the council is struggling to find the answer to. HW was not particularly happy with the absence of men, as this affects counselling sessions. But, in general, the council members were happy with the progress. The finding is consistent with a study where the authors argued that the involvement of communities in health programmes helps to advance health workers’ performance and boosts the health system [53]. The participation of HC is key to strengthening the involvement of all the health players and other non-health stakeholders in rendering health services. Nonetheless, there is a setback brought about by men’s refusal to participate in health programmes, and this may occur when there is no public information, public support, or participatory civic culture [28]. Other scholars have observed that a lack of participation may be attributed to a weak democratic framework that fails to fully incorporate people to participate actively in formal participatory platforms such as the HC [54]. A recent study in two European countries revealed that the effective implementation of innovations in local health councils requires stakeholders to develop competencies in cross-sector collaboration and adapt to unpredictable contextual influences [42]. In this regard, stakeholder involvement forms a basis for a sound healthcare system. 

## 11. Conclusions

Citizen participation in health delivery requires greater emphasis on formalised mechanisms incorporating Health Committees (HCs). The incorporation of HCs enhances citizen engagement in the administration of health amenities and service delivery. The inactivity of the HC at Itojo Hospital indicates a missed opportunity to tap into the knowledge and perspectives of citizens. This limits their influence in shaping healthcare policies and decision-making processes. The study underscored the importance of recognising citizen participation as a valuable resource in healthcare governance. By involving citizens in decision-making, health systems can benefit from their diverse experiences and insights, leading to more responsive and patient-centred healthcare services. 

Furthermore, citizen participation helps to overcome irregularity through the application of principal–agent relationships and address issues related to information control and monitoring. In the principal–agent framework in decentralised health systems, the Department of Health (DH) in local government as the principal can achieve the aims of enhanced service delivery by monitoring the performance of the agreed contract through citizen participation in Health Committees (HCs). This increases awareness of available quality services and empowers individuals to address inadequate service delivery. 

The study observes citizens’ limited influence in health delivery and recommends that health committees be integrated into the local health systems, including Itojo Hospital. Incorporating health committees into the local health systems strengthens citizen participation and fosters a sense of ownership and accountability among members of the community. This inclusive approach can lead to more effective and sustainable healthcare services that align with the needs and preferences of the people. Integrating health committees within the health system of Itojo Hospital and similar facilities can catalyse positive change, granting citizens a meaningful role in shaping the future of their healthcare. By creating avenues for citizen participation, we can move towards more patient-centred and community-driven healthcare systems that prioritise the well-being and satisfaction of the individuals they serve.

## Figures and Tables

**Table 1:** Documents and other auxiliary data analysed.

Document Type	Year	Title	Institution	Document Origin/Source/Author
Policy document report	2020	Policy Brief on Revitalising Civil Servants Performance for Sustainable Development	Cabinet secretariate	Ministry of Health Library. library.health.go.ug
Policy document report	2021	National Emergency Medical Services Policy	Ministry of Health	Ministry of Health Library. library.health.go.ug
Policy research series	2011	Local government councils’ performance and public service delivery in Uganda	Advocates Coalition for Development and Environment (ACODE) *	Advocates Coalition for Development and Environment (www.acode-u.org, accessed on 13 Jan 2024)
Policy document	2018	Community Health Extension Workers National Strategy (2018–2022)	Ministry of health	Ministry of Health Library. library.health.go.ug
News Brief	2022	Ntungamo RDC calls for improved monitoring of health services	Ntungamo District Local Government	District Website www.ntungamo.go.ug (accessed on 13 Jan 2024)
News Brief	2023	Ntungamo deputy RDC cautions health workers against absenteeism.	Ntungamo District Local Government	District Website
News Brief	2023	Ntungamo gets 1.4 billion health facility	Ntungamo District Local Government	District Website
News Brief	2024	locals tipped on utilising government health facilities	Ntungamo District Local Government	District Website
News Brief	2024	Ntungamo RDC advocates for increased HIV awareness	Ntungamo District Local Government	District Website
News Brief	2021	civil servants cautioned on absenteeism	Ntungamo District Local Government	District Website
News Brief	2021	Authorities call for collective efforts in the fight against HIV/aids	Ntungamo District Local Government	District Website
News Brief	2018	Ntungamo leaders calls for calm amidst complaints over unpaid salaries	Ntungamo District Local Government	District Website
Book: Guidelines	2018	Guidelines for Regional Referral Hospital Management Boards 2018	Uganda ministry of health	Ministry of Health Library. library.health.go.ug
Book: Guidelines	2019	Guidelines for General Hospital Management Boards 2019	Uganda ministry of health	Ministry of Health Library. library.health.go.ug
Report	2012	Report on the State of Regional Referral Hospitals in Uganda Towards the Realisation of the Right to Health	Uganda ministry of health	Ministry of Health Library. library.health.go.ug
Book: Guidelines	2016	Guidelines for Community Health Departments for hospitals	Uganda ministry of health	Ministry of Health Library. library.health.go.ug
Book: regional referral hospitals	2003	Guidelines on Hospital management boards for referral hospitals	Uganda ministry of health	Ministry of Health Library. library.health.go.ug
Book: Guidelines	2010	Guidelines on hospital management committee for district hospitals	Uganda ministry of health	Ministry of Health Library. library.health.go.ug
Book: Guidelines	2021	Guidelines for the Decentralisation of the Uganda Medical Board	Uganda ministry of health	Ministry of Health Library. library.health.go.ug
Governance report	2022	Special Report on New threats to human security in the Anthropocene 2022	UNDP	Ministry of Health Library. library.health.go.ug

Sources: Government of Uganda, departments, agencies, ministries, and private advocacy policy think tank *.

**Table 2:** Pseudo-variables with the description.

Variable	Description	Number of Respondents
HW	Health workers	10
HA	Health administrators	3
HB	Health beneficiaries (Patients/Residents)	45
LC	Local councillors	5
HCM	Health committee members	3
Total		66

Source: Author’s construct from fieldwork.

## Data Availability

Data are contained within the article.

## References

[1] Suarez-Herrera J.C., Diaz-Castro L., Ramirez-Rojas M.G., Pelcastre-Villafuerte B.E. (2024). Unpacking participation in healthcare governance: Lessons from two local health councils in Brazil and Spain. Int. J. Health Plann. Manag..

[2] McCollum R., Limato R., Otiso L., Theobald S., Taegtmeyer M. (2018). Health system governance following devolution: Comparing experiences of decentralisation in Kenya and Indonesia. BMJ Glob. Health.

[3] Masefield S.C., Msosa A., Grugel J. (2020). Challenges to effective governance in a low income healthcare system: A qualitative study of stakeholder perceptions in Malawi. BMC Health Serv. Res..

[4] Malakoane B., Heunis J.C., Chikobvu P., Kigozi N.G., Kruger W.H. (2020). Public health system challenges in the Free State, South Africa: A situation appraisal to inform health system strengthening. BMC Health Serv. Res..

[5] Kimaro H.C., Sahay S. (2007). Information Technology for Development An institutional perspective on the process of decentralization of health information systems: A case study from Tanzania An Institutional Perspective on the Process of Decentralization of Health Information Systems. Inf. Technol. Dev..

[6] Wallace L.J., Kapiriri L. (2019). Priority setting for maternal, newborn and child health in Uganda: A qualitative study evaluating actual practice. BMC Health Serv. Res..

[7] Pfeiffer J., Chapman R.R. (2010). Anthropological Perspectives on Structural Adjustment and Public Health. Annu. Rev. Anthropol..

[8] Yates R., Murindwa G., Mcpake B., Tashobya C.K., Ssengooba F., Cruz V.O. (2006). Health Systems Reforms in Uganda: Processes and Outputs.

[9] Mulumba M., Ruano A.L., Perehudoff K., Ooms G. (2021). Decolonizing Health Governance: A Uganda Case Study on the Influence of Political History on Community Participation. Health Hum. Rights J..

[10] Karuga R., Kok M., Luitjens M., Mbindyo P., Broerse J.E.W. (2022). Participation in primary health care through community-level health committees in Sub-Saharan Africa: A qualitative synthesis. BMC Public Health.

[11] World Health Organization (2021). Voice, Agency, Empowerment–Handbook on Social Participation for Universal Health Coverage. https://www.who.int/publications/i/item/9789240027794.

[12] Lodenstein E., Mafuta E., Kpatchavi A.C., Servais J., Dieleman M., Broerse J.E.W., Barry A.A.B., Mambu T.M.N., Toonen J. (2017). Social accountability in primary health care in West and Central Africa: Exploring the role of health facility committees. BMC Health Serv. Res..

[13] Mccoy D.C., Hall J.A., Ridge M. (2012). A systematic review of the literature for evidence on health facility committees in low- and middle-income countries. Health Policy Plan..

[14] Williams J.J., Cornwall A., Coelho V.S. (2007). Social change and community participation: The case of health facilities boards in the Western Cape of South Africa. Space for Change.

[15] Medard T., Yawe B.L., Bosco O.J. (2017). Health Care Delivery System in Uganda: A review. Tanzan J. Health Res..

[16] Kiiza F., Kayibanda D., Tumushabe P., Kyohairwe L., Atwine R., Kajabwangu R., Kiconco R. (2020). Frequency and Factors Associated with Hyperglycaemia First Detected during Pregnancy at Itojo General Hospital, South Western Uganda: A Cross-Sectional Study. J. Diabetes Res..

[17] Byamukama P. Financing and health service delivery in Uganda Government Hospitals: A case of Masindi General Hospital. https://umispace.umi.ac.ug/xmlui/handle/20.500.12305/826.

[18] Europe C. (2000). The Pharmacist at the Crossroads of New Health Risks—An Indispensable Partner for their Management: Proceedings, Strasbourg, 20–22 October 1999. The Pharmacist at the Crossroads of New Health Risks—An Indispensable Partner for Their Management.

[19] Onzima B. (2013). Public Accountability: Explaining Variation across Local Governments in Uganda. Master’s Thesis.

[20] Mooketsane K.S., Phirinyane M.B. (2015). Health governance in Sub-Saharan Africa. Glob. Soc. Policy.

[21] Lewis M., Pettersson G. (2009). Governance in Health Care Delivery Raising Performance. Policy Research Working Paper WPS5074 5074 Governance in Health Care Delivery Raising Performance.

[22] Bank T.W. (2015). World Development Indicators 2015.

[23] Oliveira R., Santinha G., Marques T.S. (2023). The Impacts of Health Decentralization on Equity, Efficiency, and Effectiveness: A Scoping Review. Sustainability.

[24] Martinussen P.E., Rydland H.T. (2021). Is a Decentralised Health Policy Associated With Better Self-rated Health and Health Services Evaluation ? A Comparative Study of European Countries. Int. J. Health Policy Manag..

[25] Leonardsen A.-C.L., Lappegard Ø., Garåsen H.A. (2017). Decentralised health services. J. Nor. Med. Assoc..

[26] Boadu E.S., Ile I. (2018). The politics of youth participation in social intervention programmes in Ghana: Implications for participatory monitoring and evaluation (PM&E). J. Rev. Glob. Econ..

[27] Nordjo E., Boadu E.S., Ahenkan A., Nordjo E. (2023). Community participation in enterprise development programmes for poverty reduction and sustainable development in Ghana programmes for poverty reduction and sustainable development in Ghana. Commun. Dev..

[28] De Maeseneer J., Li D., Palsdottir B., Mash B., Aarendonk D., Stavdal A., Moosa S., Decat P., Kiguli-Malwadde E., Ooms G. (2020). Universal health coverage and primary health care: The 30 by 2030 campaign. Bull. World Health Organ..

[29] Franchini M., Salvatori M., Denoth F., Molinaro S., Pieroni S. (2022). Participation in Low Back Pain Management: It Is Time for the To-Be Scenarios in Digital Public Health. Int. J. Environ. Res. Public Health.

[30] Haustein E., Lorson P.C. (2023). Co-creation and co-production in municipal risk governance—A case study of citizen participation in a German city. Public Manag. Rev..

[31] World Health Organization (2006). WHO Constitution on the Right to Health and the Role of the State in Ensuring This Right Is Attainable. The Constitution Affirms the Importance of Informed Opinion and Cooperation of Citizens in Achieving Health Gains. https://www.who.int/about/accountability/governance/constitution.

[32] Dean H. (2008). Social policy and human rights: Re-thinking the engagement. Soc Policy Soc..

[33] Chancel L., Cogneau D., Gethin A., Myczkowski A., Robilliard A.S. (2023). Income inequality in Africa, 1990–2019: Measurement, patterns, determinants. World Dev..

[34] Eusebio C., Bakola M., Stuckler D. (2023). Commentary How to Achieve Universal Health Coverage: A Case Study of Uganda Using the Political Process Model. Kerman Univ. Med. Sci..

[35] Mukuru M., Gorry J., Kiwanuka S.N., Gibson L., Musoke D. (2022). Designed to Fail ? Revisiting Uganda’s Maternal Health Policies to Understand Policy Design Issues Underpinning Missed Targets for Reduction of Maternal Mortality Ratio (MMR): 2000–2015. Int. J. Health Policy Manag..

[36] Oluwafemi P., Fajobi O., Oluwatobi T., Olaniyan M.E., Abdulazeez O., Blessing O., Oko C. (2022). Healthcare systems strengthening in Africa: The call for action to achieve SDG 3. Int. J. Health Plann. Mgmt..

[37] Batley R., Mcloughlin C. (2015). The Politics of Public Services: A Service Characteristics Approach. World Dev..

[38] Golooba-mutebi F. (2005). When Popular Participation Won’t Improve Service Provision: Primary Health Care in Uganda. Dev. Policy Rev..

[39] Devarajan S., Widlund I. (2007). The Politics of Service Delivery in Democracies: Better Access for the Poor.

[40] Pratt J.W. (1985). Principals and Agents: The Structure of Business.

[41] Hedge D.M., Scicchitano M.J., Metz P. (1991). The Principal-Agent Model and Regulatory Federalism. West Polit. Q..

[42] Kumar K. (1989). Conducting Key Informant Interviews in Developing Countries (p. 1). Washington DC: Agency for International Development. [Internet]. Vol. 1, NBER Working Paper Seriesking Paper Series. https://www.unhcr.org/publications/manuals/4d9352319/unhcr-protection-training-manual-european-border-entry-officials-2-legal.html?query=excom.

[43] Mariotto F.L., Zanni P.P., Salati G.H., de Moraes M. (2014). What is the use of a single-case study in management research?. Rev. Adm. Empres.

[44] Wiersma W. (2000). Research Methods in Education: An Introduction.

[45] Patton M.Q. (2002). Qualitative Evaluation and Research Methods.

[46] Bowen G. (2009). Document Analysis as a Qualitative Research Method. Qual. Res. J..

[47] Bossert T. (1998). digitalization and rural health delivery in ghana: Opportunities, challenges, and implications 1. introduction digitalization has the potential to revolutionize rural health delivery in ghana by improving access, efficiency, and quality of healthcare serv. Soc. Sci. Med..

[48] Clarke V., Braun V. (2017). Thematic analysis. J. Posit. Psychol..

[49] Lincoln Y.S., Guba E.G. (1986). But Is It Rigorous? Authenticity in Trustworthiness and Naturalistic Evaluation. New Dir. Progr. Eval..

[50] Kusnanto H. (2001). Principal-Agent and Stakeholder Approaches in Decentralized Health Care: The Indonesian Case.

[51] Donaldson S.I., Lipsey M.W., Shaw I., Greene J., Mark M. (2001). Roles for theory in evaluation practice. Handbook of Evaluation.

[52] Laffont J.J., Martimort D. (2001). The Theory of Incentives I: The Principal-Agent Model.

[53] Lambert H., Mckevitt C. (2002). Anthropology in health research: From qualitative methods to multidisciplinarity. BML.

[54] Bailey A., Mujune V. (2024). Multi—Level change strategies for health: Learning from people—Centered advocacy in Uganda. Int. J. Equity Health.

[55] Zhang Y., Wildemuth B., Wildemuth B.M. (2017). Qualitative Analy sis of Content. Applications of Social Research Methods to Questions in Information and Library Science.

[56] Berg B.L. (2001). Qualitative Research Methods for the Social Sciences.

[57] Bradley J. (1993). Methodological Issues and Practices in Qualitative. Symposium on Qualitative Research: Theory, Methods, and Applications.

[58] Nabyonga-orem J., Karamagi H., Atuyambe L., Bagenda F., Okuonzi S.A., Walker O. (2008). Maintaining quality of health services after abolition of user fees: A Uganda case study. BMC Health Serv. Res..

